# Supplementary material for: Conversion of a conventional superconductor into a topological superconductor by topological proximity effect
Source: Nat Commun. 2020 Jan 9;11:159. doi: 10.1038/s41467-019-13946-0 (PMC6952357; doi:10.1038/s41467-019-13946-0)
Supplement: Supplementary file 1 — Supplementary Information [file 41467_2019_13946_MOESM1_ESM.pdf]

SUPPLEMENTARY INFORMATION for

Conversion of a conventional superconductor into a topological superconductor by topological proximity effect

Trang et al.

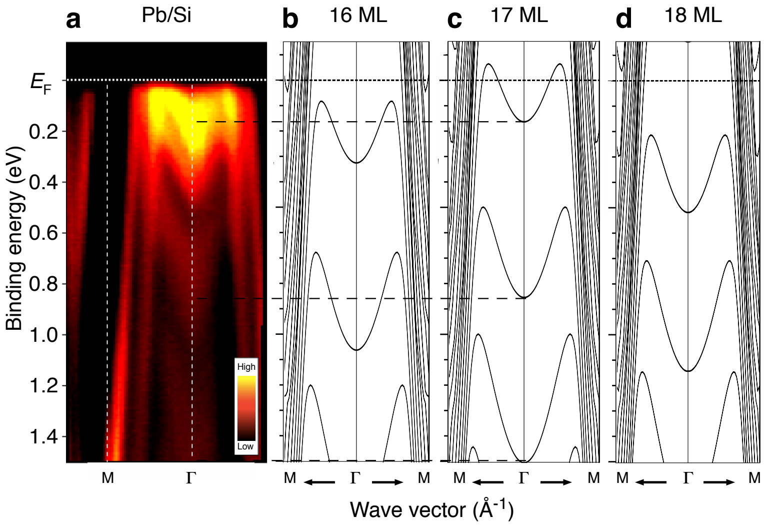

Supplementary Figure 1. Comparison of band dispersion of Pb between ARPES and band calculations. a Plot of ARPES intensity measured along the $\overline{\Gamma M}$ cut for 17 ML Pb film on Si(111). **b-d** Calculated band structures along the $\overline{\Gamma M}$ cut for 16, 17, and 18 MLs of Pb slabs, respectively.

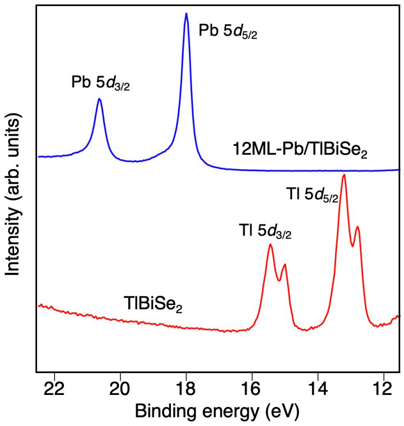

Supplementary Figure 2. Comparison of ARPES spectrum around the Tl 5*d* and Pb 5*d* core-level region between pristine $TlBiSe_2$ and 12ML-Pb/$TlBiSe_2$ measured with the He IIα photons ($h\nu$ = 40.8 eV).

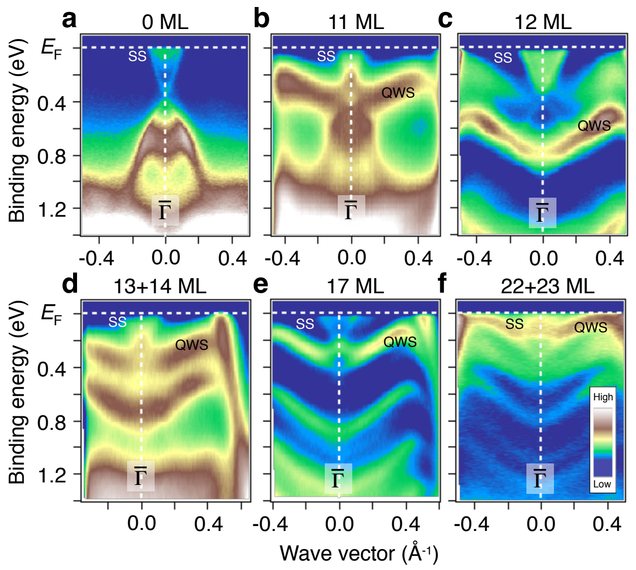

Supplementary Figure 3. Film-thickness dependence of experimental band structure in Pb/TlBiSe₂. a-f Plots of ARPES intensity of Pb(111)/TlBiSe₂ measured along the $\overline{\Gamma K}$ cut for various film thickness *n* (*n* = 0, 11, 12, 13+14, and 22+23 ML, respectively). 13+14 ML represents the spatially inhomogeneous Pb film in which 13-ML and 14-ML islands are formed on TlBiSe₂..

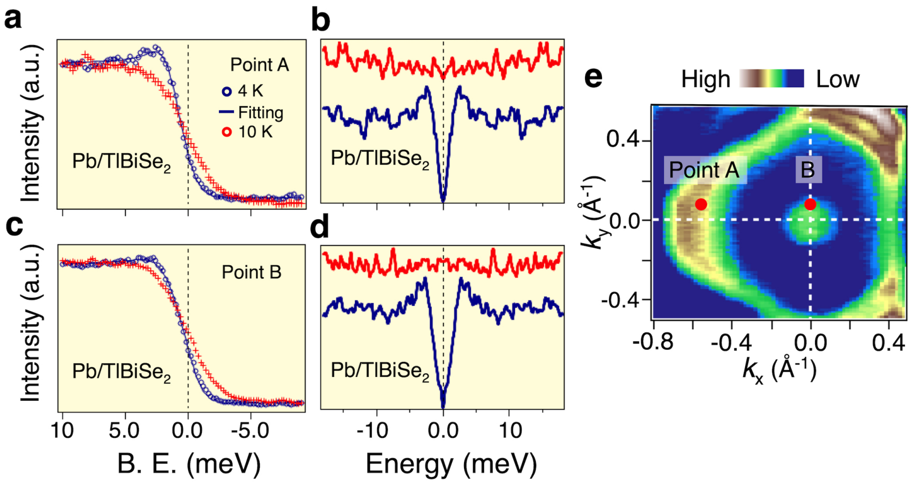

Supplementary Figure 4. Observation of superconducting gap in 12ML Pb/TlBiSe₂. **a**, **b** Ultrahigh-resolution EDCs and corresponding symmetrized EDCs, respectively, at T = 4 K and 10 K measured at point A on the Fermi surface shown in **e**, for 12 ML Pb film on TlBiSe₂. **c**, **d** Same as (a, b), respectively, but measured at point B in (e). **e** Mapping of ARPES intensity near E_F and measured $\mathbf{k}_F$ points (points A and B).

Supplementary Note 1: Estimation of the film thickness using the band-structure calculations

We have estimated the number of monolayers (MLs) of Pb films by comparing the energy location of the quantum well states (QWSs) between the ARPES results and the slab calculations for free-standing *n*-ML Pb film (*n* = 10-24). Supplementary Figure 1 shows a comparison of band structure along the $\overline{\Gamma M}$ cut between the ARPES result for a 17 ML Pb film on Si(111) and corresponding band structures calculated for free-standing 16-18ML films. Both the ARPES and the calculations commonly show a M-shaped feature, suggesting that this feature originates from the QWSs of Pb film. The energy location of the QWSs in the experiment for a 17 ML Pb film shows a reasonable agreement with that of the calculation for 17 ML (Supplementary Figure 1c), whereas the calculations for 16 or 18 ML show a deviation from the ARPES result. This suggests that our way of estimating the film thickness has an accuracy within 1 ML. We have applied the same method for all Pb films fabricated on $TlBiSe_2$ or Si(111) to estimate the film thickness. We confirmed that the estimated film thickness is consistent with that estimated from the Pb-deposition time/rate during the molecular-beam epitaxy. It is noted that there exists a small quantitative difference in the energy position of QWSs between the slab calculations and the ARPES results (e.g., ~ 50 meV at the Γ point for the second QWSs). This may be understood in terms of the substrate effect related to the interfacial phase shifts.

Supplementary Note 2: Change in the core-level peaks upon evaporation of Pb on $TlBiSe_2$

We measured the ARPES spectrum in a wide energy region covering the Tl 5*d* and Pb 5*d* core-level region before/after evaporation of Pb on $TlBiSe_2$. As shown in Supplementary Figure 2, the Tl 5*d* core-level peaks located at the binding energy E_B of ~12-16 eV completely disappear after depositing a 12ML Pb film on $TlBiSe_2$, while the Pb 5*d* core-level peaks simultaneously evolve at ~17.5-21 eV. This suggests that the surface measured by ARPES is fully covered with Pb, and the observed Dirac-cone feature in Pb/$TlBiSe_2$ indeed migrates from the surface of $TlBiSe_2$. Absence of Tl peak

after the evaporation of Pb might suggest a clean interface/surface nature, whereas a possibility of Se segregation is not completely excluded since the energy range of the measurement does not cover Se core levels.

Supplementary Note 3: Thickness dependence of the band structure of Pb thin films on $TlBiSe_2$

We observed the Dirac-cone energy band hybridized with the QWSs in the Pb thin film on $TlBiSe_2$ over a wide range of film thickness (n = 11-22 ML). We show in Supplementary Figure 3 the film-thickness dependence of ARPES intensity measured along the $\overline{\Gamma K}$ cut. One can clearly recognize the QWSs whose energy position systematically changes on increasing the number of layers n. One can also identify an additional feature above the topmost QWSs, which is assigned to the Dirac-cone band migrating from the $TlBiSe_2$ surface. We found that the energy location of the Dirac-cone band, as well as its sharpness and intensity, systematically changes with the film thickness. Moreover, the Dirac-cone band looks as if it is heavily hybridized with the topmost QWSs, producing a complicated intensity profile around the intersection of the Dirac-cone band and the QWSs.

Supplementary Note 4: Superconducting-gap measurement for the 12 ML Pb thin film

We observed a superconducting gap in the Pb thin film on $TlBiSe_2$ at least down to the film thickness of 12 ML. Supplementary Figure 4a-d shows the EDCs and symmetrized EDCs at two representative Fermi wave vectors ($\mathbf{k}_F$) on the Pb-derived triangular Fermi surface and the Dirac-cone band migrating from $TlBiSe_2$ (points A and B in Supplementary Figure 4e, respectively) measured at T = 4 and 10 K. Similarly to 17ML-Pb/$TlBiSe_2$ (Fig. 3d-i in the text), we observe a small pile up in the spectral weight accompanied with a leading-edge shift for both points, indicative of the superconducting-gap opening. We confirmed that this spectral change is due to the superconducting-gap opening, since such spectral feature is absent at T = 10 K above T_c of bulk Pb (7.2 K). We

have estimated the magnitude of superconducting gap to be 1.2 ± 0.1 meV for both $\mathbf{k}_F$ points by the numerical fittings using the Dynes function multiplied by the Fermi-Dirac distribution convoluted with a resolution function. This value is similar to those estimated for the 17 and 22 ML films, suggesting that the T_c value of our Pb films maintains the constant value similar to bulk Pb at least down to 12 ML.

We found that the coherence peak in the 22 ML film (Fig. 3b in the text) is better seen than the 17 ML (Fig. 3d in the text) and 12 ML (Supplementary Figure 4a,b) films. This is also reflected in the broadening factor (damping parameter Γ) in the numerical fittings with Dynes function at point A on the Pb-derived Fermi surface (0.2, 0.8, and 0.5 meV for the 22-, 17-, and 12-ML films, respectively). While we do not know an exact origin for such a difference, we speculate that the surface quality, such as inhomogeneity and domain size of Pb film, is somehow related to the quasiparticle scattering rate. This conjecture could be further examined by elucidating the relationship between the surface structure and the superconducting gap with low-temperature scanning tunneling microscopy.
